# Inflammation of the Vena Portæ (Pylephlebitis)

**Published:** 1848

**Authors:** John Waller

**Affiliations:** Prague


THE NORTH-WESTERN

### MEDICAL AND SURGICAL JOURNAL.

Vol. I.] APRIL AND MAY, 1848. [No. 1.

### Part 1.—Original Communications.

### ARTICLE I.

*Inflammation of the Vena Portæ* (*Pylephlebitis*). By Dr. John Waller, of Prague. Translated from the German, by Daniel Stahl, M.D., of Quincy, Illinois.

The author, before entering upon the subject of his treatise, details the history of five cases of inflammation of the vena portæ, with their post mortem appearances. These cases are very minutely and too lengthily narrated to fit them for the narrow limits which this Journal prescribes for non-original matter. The translator has therefore deemed it prudent to communicate the results which the author has drawn from his and other cases; and it is hoped that this sketch may give an impulse to our American medical practitioners to direct their observations to a disease which, although hitherto overlooked, cannot be rare in a country where diseases of the portal system are as prevalent as they are in the western and southern parts of America.

*Anatomical Character.*—Referring to what *Rokitansky* has said in his Treatise on Inflammation of the Vena Portæ, the author confines himself to the results drawn from the cases detailed.

1. The changes in the parieties of the vena portæ, were, in all the cases, trifling, and stood in no proportion to the morbid changes of the contents of the vena. The circumstance, that in one case the parieties of the superior mesenteric vein were whitish-gray, shrivelled, obliterated, and almost cartilaginous in some places, renders it probable that the inflammatory action proceeded from thence. The lumen of the tube of the vein was, in all cases, dilated by the products of inflammation contained in its canal, the trunk of the vein was very much distended by its contents, and its morbid condition perceptible by the eye from its external appearance.

2. Changes in the blood itself of the vena portæ; (*a*) blood coagulations of different degrees were found in four cases; (*b*) pus was absent in three cases; (*c*) acute medullary sarcomatous depositions were found in two cases. Concerning the extent of the inflammatory action in the portal system, it was found that in the first cases only was the spleen free; in the second and fifth were the branches of the vena portæ in the liver, its rami and trunk, but not the splenic and mesenteric vein; in the third case, the splenic vein, one branch of the inferior mesenteric vein, the trunk to its finest ramifications; in the fourth case, the vena portæ in its whole extent, implicated in the morbid process. That we have to deal, not with a primary but a secondary phlebitis, called forth by bloody coagulation, is evident, among other reasons, from the entire absence of anatomical changes in the parieties of the vena portæ, whilst the marks of inflammation of its contents are so obvious. The coagulation of blood was, in all cases, the first change, no matter whether this took place spontaneously or whether it was produced by pus or its elements. In these coagulations, takes place partly an imperfect, partly a perfect excretion (or separation) of fibrini in the canal of the vein; the longer the disease continued the more complete was the change of the lymph. Coagulations with pus, as a sequence to the suppurative phlebitis, manifested itself in the first secondary inflammation of the parieties of the superior mesenteric veins; in the other cases, death took place before any sequelæ could develop themselves. The time within which, after the coagulation of the blood, inflammation of the parieties of the vein can take place, can be various. Concerning the duration of the disease, there appeared, in the first case, even after the forty-seventh day of sickness, no inflammation of the vena portæ, with the exception of one part of the superior mesenteric vein; whilst in the fourth case it was observed in the course of four days. It appears, therefore, that the acuteness and the intensity of the disease of the blood exercise a certain influence.

*Symptomatology.*—To find the vena portæ and its branches, by means of physical examination is, by their anatomical position, impossible; we must, therefore, depend on the other phenomena observed in the reported cases, and here we have, before all others, to consider the *objective local symptoms.* Among these is the abdominal distention, which is caused either by meteorism or accumulation of serum, the most prominent. Neither meteorism nor ascites appear to have diagnostic value in pylephlebitis, because there can, in no case, be shown a direct connection of it with inflammation of the vein; meteorism depended on the pyæmia and on the peritonitis, which existed simultaneously; the ascites depended on cancer and granulation of the liver. The size of the liver was enlarged in all the five cases. The cause of this enlargement was—in the first two cases, besides numerous abscesses in the liver, a distention, with pus and plastic coagulations, of the ramifications of the vena portæ; in the third case a large quantity of pus in the branches alone of the vena portæ; in the fourth and fifth cases, besides the blood coagulations and cancerous depositions in the portal ramifications, and abundant cancerous deposition in the substance of the liver. It follows from this that, although the increase of the volume of the liver is intimately connected with inflammation of the portal ramifications, we cannot, from this symptom, infer, with certainty, the existence of this disease, because many other diseases have the same symptom. Icterus was observed in four cases—in the first two and the last two; it was not observed in the fifth case. In the first and second cases were found numerous abscesses in the liver, which contained pus mixed with bile. In the fourth case was granulated liver and sarcoma, the cause of the icterus. In the last case, the hue of the skin was such as is observed in scirrhous dyscrasia. Now, whether the jaundice hue of the skin arose in consequence of pylephlebitis without abscesses of the liver or not, in none of the cases related can jaundice be the consequence of pyæmia alone; jaundice cannot, therefore, be regarded as a pathognomic symptom. Enlargement of the spleen has been mentioned already by *Schoenlien* as a diagnostic symptom, and was found in four of our cases. Anatomy informed us of mechanical hyperæmia as the cause of this enlargement in the first three cases, to which, in the second case, was added partial plastic exudation; in the third case, moreover, an abundant secretion of pus in the smallest beginning and in the trunk of the splenic vein. In the fourth case the enlargement was deducible from granulation and scirrhus of the liver; and in the fifth case, at last, was the spleen even smaller—its skeleton, however, hypertrophied. Enlargement of the spleen can obviously be produced by inflammation of the vena portæ, and furnishes, in connection with the other symptoms, no unimportant diagnostic sign in those cases where a minute examination of the size of the spleen by means of pecussion is permitted by the condition of the thoracic and abdominal organs. *Balling* and *Schoenlein* mention nausea, vomiting, and even vomiting of blood, as symptoms. The author observed in but three of the recorded cases vomiting of whitish or yellowish fluid; it was occasioned in one case by encephalitis; in another by chronic catarrhal affection of the stomach and pressure of the scirrhous liver upon the stomach; in the third case, where vomiting took place but once, was no sufficient explanation by a post mortem examination discovered. Vomiting can, therefore, in the cases recorded, not be considered as a symptom of pylephlebitis. The alvine evacuations are seldom normal; they were tardy in the first case; sero-mucous diarrhæ, which was at times deep green, tormented the patient of the second case; constipation was in the third; then yellow, greenish discharges, which were sometimes mixed with streaks of blood, appeared in the fourth case; and in the fifth were muco-serous or dark, soft evacuations. Only the third case showed in the mucous membrane of the small and large intestinal canal partial acute catarrh; in the fourth case, chronic catarrh in the same parts and also in the stomach—the intestinal canal of the other patients showed no particular abnormity. The diarrhæa of the second patient must, therefore, be considered as a symptom of pyæmia ; and the constipation of the third must be ascribed to encephalitis. The chronic catarrh of the fourth, and the dark, ruft evacuations of the fifth, existed prior to the advent of the phlebitis. The variously constituted alvine evacuations, as well as the hæmoptysis, can accompany peryphlebitis ; yet they never stand in immediate connection together, but manifest themselves only by means of the mechanical hyperæmia of the intestinal mucous membrane or of other complications. The distention of the venæ epi and hypogastricæ, produced by mechanical hyperæmia, which *Schoenlein* has defended as a symptom of our phlebitis, has not been observed by the author. Pain, the *subjective local symptom*, was not at all present in the fourth case, was experienced by times, and increased on pressure in the first case, and in the second case was the whole epigastrium sensitive, the inguinal iliac regions very painful ; in the third and fifth cases was pain also severe. In all of these cases existed complications which, besides the inflammation of the vena portæ, caused pain. It is, therefore, not certain whether the inflammation of the vena portæ is accompanied by pain or not. Ascites has, by *Cruveilheir*, been considered as a symptom of pylephlebitis ; neither the author nor other observers had opportunity to confirm this ; sure it is, that obliteration of the vena portæ, by whatever cause produced, has ascites as a consequence. The œdema of the lower extremities, which was present in cases No. four, and five, (in the two last already prior to the inflammation of the vena portæ) and was absent in cases No. one and three, is, for anatomical reasons, not to be regarded as depending on inflammation of the vena portæ ; because the veins of the lower extremities lose themselves, not in the vena portæ, but in the vena cava inferior. Occasional pulsation of the right jugular vein, pulsation in the abdomen and in different places of the body, fainting, dry cough and the like, which *Balling* regarded as symptoms of this disease, cannot be regarded as such by the author, because he himself has neither observed them nor can he explain their connection with the disease. In relation to *the functions of the vascular system*, Baczynski thinks to have observed in the inflammatory stage the symptoms characterizing inflammatory fever, and in the second stage a sinking (depression) of the whole nervous and vascular system; *Schoenlein* boasts to have recognized, by a rigor, the transition of an inflammation from the peritoneum to the vena portæ. The possibility that inflammation of the vena portæ may be accompanied by an inflammatory fever, cannot be denied; the observations, which are before us, establish only that form of fever which has heretofore been recognized as adynamic or nervous; this form of fever proves, in our cases, only the existence of pyæmia. In the second and third of the detailed cases seems to exist no doubt but what the advent of the inflammation of the vena portæ was announced by rigor; and even in the fourth and fifth cases was rigor the first symptom of the inflammation of the vena portæ. The pulse was, throughout, accelerated, and beat ninety-two to one hundred and twenty times in a minute. The skin, was, in every case very hot; in the first, at first dry, afterwards profuse perspiration, as was the case in all the other cases, and in two cases there were miliariæ; in the fourth case, petechiæ. In the mucous membranes we find in the first case pulmonary catarrh; in the second and third cases croup (?) of the mucous membrane of the fauces as consequences of pyæmia; and also in the third case acute catarrh of the small and large intestines from pyæmia. General peritonitis followed plainly in consequence of pyæmia in the third case. The pyæmia manifested its deleterious influence in the reproductive sphere by a speedy disappearance of fat and muscular substance; it caused but slight deviation in the nervous system; the cerebral symptoms in the third case, must be ascribed to the encephalitis. Two of the patients showed a peculiar gloomy expression, apparently emanating from deep suffering. If, therefore, some observers mention delirium, stupor, distortion of features, wakefulness or coma as symptoms of inflammation of the vena portæ, these symptoms can, according to the author's observations, be deduced from pyæmia. Particularly worthy of regard, as symptoms, are the enlargement of the liver, jaundice, pyæmia, and enlargement of the spleen; but, supported even by these, the diagnosis cannot be positive.

*Duration, Course, and Termination.*—The shortest time of illness in the recorded cases, was three, and the longest, forty seven days. According to this difference of duration, has this disease been distinguished into acute and chronic; but this is unimportant. Likewise has the acute been divided into an inflammatory and adynamic stage ; as cause of the adynamic condition was, in all cases, pyæmia observed. The termination of inflammation of the vena portæ in health cannot be doubted ; but has as yet not been observed. The most frequent termination was in suppuration, which, to recognize at the sick bed, seems to be difficult. A consequence of the suppurative pylephlebitis is the formation of abscesses in the liver ; adhesion and obliteration take place in the trunk, or, more frequently, in the branches and rami of the vena portæ ; this termination manifests itself by enlargement of the spleen, hyperæmia of the mucous membrane of the intestinal canal and stomach, swelling of the hemorrhoidal veins, hyperæmia of the peritoneum, ascites, &c., &c.—all symptoms which can be traced to mechanical hyperæmia and its consequences. The most frequent termination, death, took place in all the cases observed ; in our cases it was caused partly by pyæmia, partly by scirrhous dyscrasia, and scirrhous degeneration of the stomach and of the liver. Complications, also, can cause death. Some were cause, others consequence of pyæmia ; others were caused by scirrhous discrasia ; ascites and anasarca were consequences of granulation of the liver and of scirrhous of the liver and its krasis.

*Ætiology.*—The author considers the case, observed and described by *Balling*, as primary pylephlebitis ; there was to be found, neither in the cadaver nor at the sick bed, a cause of the disease, and the diseased condition reached in the tunics of the blood vessels to a higher degree, and seemed to be of an older date, than in the blood itself. As causes of the primary pylephlebitis, are to be mentioned traumatic influences and cold. Almost all the cases of inflammation of the vena portæ, which have, as yet, been observed, are secondary, i. e. the coagulation of the diseased blood is the primary and most essential occurrence, after which inflammation of the tunics of the veins is developed. That this has also been the case in the five cases recorded, is shown by the author in detail. According to the observations of others, pylephlebitis appears also frequently as secondary disease ; it is developed in consequence of inflammation of the substance of the liver, abscesses of the liver, of the biliary ducts, of the spleen, or in consequence of the inflammation of other veins. We know nothing certain as to a peculiar predisposition to pylephlebitis ; it may occur frequently in the newborn, on account of the frequency of inflammation of the umbilical vein at that age, which inflammation is then easily transmitted to the vena portæ. We know nothing as to the influence of age and sex. We cannot deny that the prognosis must generally be unfavorable. As long as the diagnosis is doubtful, there can nothing be said of the treatment.

The above account has been translated from "Schmidt's Jahr. Bucher der in—and auslandischen Jesammten medicin," No. 8, 1847. This is, consequently, the latest inquiry on record into this rare disease. As the translator can find hardly any mention of inflammation of the vena portæ in any of the usual works on the practice of medicine, and as the observations are not sufficiently numerous yet to have any certain diagnosis, &c., of the same, and as, moreover, there is a discrepancy in relation to the pathology, symptomatology, &c., of the cases already published, he has considered it a service to the profession of this country, to translate also the leading features of the remarks on this disease, which are contained in the "Encycklopœdie der Gesammten Medicin von Carl Chr. Schmidt, Med. and Chir. Doctor," vol. 6., Leipzig, 1842.

"*Inflammation of the vena portæ*" says Dr. Eisenmann in the above work, "occurs upon the whole but rarely, yet there are a goodly number of observations at our command, namely, a case by *Bouilland*,* two by *Reynaud*,† one by *Dance*,‡ one by *Bonie*,§ one by *Balling*,|| one by *Schoenlein*,¶ two by *Andral*,** one by *Aullier*,†† one by *Cruveilhier*,‡‡ and one by *Mohr*.§§ *Schoenlein* has also recently observed a case at Berlin. It is

* Archiv. Gener. 1823, Juin.

† Journ. Hebdom. II, and Revue Med. 1839.

‡ Archiv. Gener. 1828, Dec., 1829, Feb.—20 observations.

§ Clinique des Hopitan, 1829, May.—Bullet. des Sciences Med. xvi, 216.

|| Zur Venenentzundung, page 310.

¶ *Baczynski* Diss. de Venæ Portarum Inflammatione, Zurich, 1838. Schmidt's Jahrb. xxii, 110.

** On these four cases compare *Baczynski's* Dissert.

†† Central Zeitung, 1840, Nov. 29. Schmidt's Jahrb. xxii, 110.

‡‡ On these four cases compare *Baczynski's* Dissert.

§§ Gentral Zeitung, 1840, No. 29. Schmidt's Jahrb. xxviii, 50.

also to be noticed, that those cases of inflammation of vena portæ are not included which are observed in newborn infants, where the inflammation proceeded from the umbilical vein.

The inflammation of the vena portæ can, like that of other veins, proceed from the inside of the vein and extend to the outside of the same; but the inflammation can also originate in the surrounding tissues, and extend itself to the external and from thence to the internal membrane of the vena portæ; and if, in *Reynaud's* case, the vena portæ and cava were closed externally by exæudation; if in *Aullicr's* was found, in the vena portæ, blood of the consistence of cream, but in the liver, abscesses, and other vestiges of inflammation; if, finally, in *Cruveilheir's* case the cellular tissue of the vena portæ was infiltrated with pus; then we can well assume, that in these cases the inflammation proceeded from without to within, whilst in the cases of *Dance*, *Bonic*, *Balling*, and others, the course of development of the disease took its direction from within to without.

The anatomical changes and the physiological anomalies are the same with the vena portæ as with inflammation of other veins,* and it is added to it only the functional derangements of the vena portæ and of the liver. This inflammation can take an acute and a chronic course. *Baczynski* deduces from nine cases, the following picture of the acute inflammation of the vena portæ: (*a*) Inflammatory stage, local symptoms: sudden pain in the regio epigastrica and in the hypochondrium dextrum, without (?) obvious causes; this pain returns like colic, increases on pressure very much, and extends itself gradually over the whole abdomen; heat, deep (low?) in the hypochondrium; the abdomen is dense, full, or in the beginning retracted, or only moderately swelled; hard, but is not meteoric; veins of the abdominal parieties distended, extending thus to the chest and axilla. General symptoms: fever, which takes place sometimes before, sometimes with, and sometimes after the local symptoms; severe chill, (rigor) which lasts from one to two hours and is followed by heat; quick, soft, weak, and small pulse;

*Compare with this Dr. Waller's opinion on pages 1 and 2. Transl.

dry and turgescent skin; tongue dry and covered back with thick, white mucous; abdomen very warm; thirst and red fauces, continued heaviness of the head, vertigo, debility, anxiety and restlessness of mind, wakefulness, constipation, scanty, red urine with a sediment like brickdust. After a while appear difficulty of breathing, tossing, and an icteric hue of the skin. (*b*) Adynamic stage: it commences from the third to the eighth day of the disease. The first stage never lasts to the fourteenth day. The progress of the disease is frequently very obscure. Local symptoms: the pain and heat of the abdomen disappear entirely; a throbbing sensation in the abdomen, and sometimes in other parts of the body. The abdomen swells more. General and sympathetic symptoms: retching, vomiting of a black substance resembling dissolved soot, pressure on the abdomen produces stitches and retching, palpitation, irregular and unequal pulsation of the heart, great anxiety, the face is distorted and covered with perspiration, mouth, nostrils, and the trembling tongue, have a dark color, great debility, unconsciousness, anxious, staring eyes, dilated pupil, which was contracted, sopor, aoma with delirium, automatic motion of the hand to the abdomen, very small pulse, which cannot be counted any more. A great deal of dark blood runs from the anus shortly before death, the strength continues sinking, the countenance becomes hippocratic, the extremities are cold, and death takes place in the course of seven days."

*Baczynski* has either not observed, or has *omitted* to notice, the *chill* or *rigor* which announces the commencing suppuration.

"The *chronic form*, as described by *Baczynski*: Disagreeable weight in the scrobiculum cordis, a feeling as if every thing in the abdomen were arrested in its function. Little heat in the region of the liver; pain in that region from the commencement; abdomen somewhat swelled and painful on pressure: yellow hue of the skin; distention of the veins of the abdomen and extremities, then feeble febrile excitement, in the evening frequent alternations of chilliness and febrile heat; skin dry, temperature little elevated; pulse quick and soft, and again slow and weak. Dyspeptic symptoms, vomiting of dark green matter, mixed with blood; tardy stools; urine scanty, red, and with brick-dust sediment; oppression of the chest, hawking. Abdominal distension and emaciation increases. Sometimes œdema. Strength diminishes, and the emaciation progresses, notwithstanding there is at times a good appetite. Hectic. Mild delirium. Death. The disease lasts from a month to a year."

We know but little, as yet, of the cause of primary pylephlebitis. "More frequent is secondary pylephlebitis, and this can arise in three different ways: (*a*) an inflammation of the liver can communicate itself to the vena portæ and penetrate its membranes; (*b*) an inflammation of the stomach, spleen intestinal canal, can penetrate to the roots of the vena portæ and can reach into the trunk and further; (*c*) deleterious substances, pus, or ichor can be absorbed in the stomach, intestinal canal, or spleen, and cause inflammation in the vena portæ. In the case observed by *Bonie*, there were syphilitic affections of the rectum and the bladder; the trunk of the vena portæ, from the place where it enters the liver to its last ramifications, was full of white, thick pus, and the walls were covered with pseudo-membranes. Could not here the pylephlebitis be produced by syphilitic pus?

The treatment of the inflammation of the vena portæ is similar to that of phlebitis generally. The secondary, however, requires a rational treatment of the primary affection; and against the suppurative fever, if it should manifest itself, should be instituted a disinfecting and conservative treatment.

---

